# An updated 18S rRNA phylogeny of tunicates based on mixture and secondary structure models

**DOI:** 10.1186/1471-2148-9-187

**Published:** 2009-08-05

**Authors:** Georgia Tsagkogeorga, Xavier Turon, Russell R Hopcroft, Marie-Ka Tilak, Tamar Feldstein, Noa Shenkar, Yossi Loya, Dorothée Huchon, Emmanuel JP Douzery, Frédéric Delsuc

**Affiliations:** 1 Université Montpellier 2, Institut des Sciences de l'Evolution (UMR 5554), CC064, Place Eugène Bataillon, 34095 Montpellier Cedex 05, France; 2 CNRS, Institut des Sciences de l'Evolution (UMR 5554), CC064, Place Eugène Bataillon, 34095 Montpellier Cedex 05, France; 3 Centre d'Estudis Avançats de Blanes (CEAB, CSIC), Accés Cala S. Francesc 14, 17300 Blanes (Girona), Spain; 4 Institute of Marine Science, University of Alaska Fairbanks, Fairbanks, Alaska, USA; 5 Department of Zoology, George S. Wise Faculty of Life Sciences, Tel Aviv University, Tel Aviv, 69978, Israel; 6 Department of Biology, University of Washington, Seattle WA 98195, USA

## Abstract

**Background:**

Tunicates have been recently revealed to be the closest living relatives of vertebrates. Yet, with more than 2500 described species, details of their evolutionary history are still obscure. From a molecular point of view, tunicate phylogenetic relationships have been mostly studied based on analyses of 18S rRNA sequences, which indicate several major clades at odds with the traditional class-level arrangements. Nonetheless, substantial uncertainty remains about the phylogenetic relationships and taxonomic status of key groups such as the Aplousobranchia, Appendicularia, and Thaliacea.

**Results:**

Thirty new complete 18S rRNA sequences were acquired from previously unsampled tunicate species, with special focus on groups presenting high evolutionary rate. The updated 18S rRNA dataset has been aligned with respect to the constraint on homology imposed by the rRNA secondary structure. A probabilistic framework of phylogenetic reconstruction was adopted to accommodate the particular evolutionary dynamics of this ribosomal marker. Detailed Bayesian analyses were conducted under the non-parametric CAT mixture model accounting for site-specific heterogeneity of the evolutionary process, and under RNA-specific doublet models accommodating the occurrence of compensatory substitutions in stem regions. Our results support the division of tunicates into three major clades: 1) Phlebobranchia + Thaliacea + Aplousobranchia, 2) Appendicularia, and 3) Stolidobranchia, but the position of Appendicularia could not be firmly resolved. Our study additionally reveals that most Aplousobranchia evolve at extremely high rates involving changes in secondary structure of their 18S rRNA, with the exception of the family Clavelinidae, which appears to be slowly evolving. This extreme rate heterogeneity precluded resolving with certainty the exact phylogenetic placement of Aplousobranchia. Finally, the best fitting secondary-structure and CAT-mixture models suggest a sister-group relationship between Salpida and Pyrosomatida within Thaliacea.

**Conclusion:**

An updated phylogenetic framework for tunicates is provided based on phylogenetic analyses using the most realistic evolutionary models currently available for ribosomal molecules and an unprecedented taxonomic sampling. Detailed analyses of the 18S rRNA gene allowed a clear definition of the major tunicate groups and revealed contrasting evolutionary dynamics among major lineages. The resolving power of this gene nevertheless appears limited within the clades composed of Phlebobranchia + Thaliacea + Aplousobranchia and Pyuridae + Styelidae, which were delineated as spots of low resolution. These limitations underline the need to develop new nuclear markers in order to further resolve the phylogeny of this keystone group in chordate evolution.

## Background

For more than a century, it has been known that tunicates (or urochordates) belong to chordates [1]. Traditionally occupying a basal position within chordates, they quickly became model organisms in evolutionary developmental studies aimed at understanding the origins of chordates. The complete genome sequence of the ascidian *Ciona intestinalis *revealed the essential toolkit of vertebrate genes in a small and compact genome that had not undergone the round of vertebrate-specific genome duplication events [2,3]. However, the *Ciona intestinalis *genome has also been evolving rapidly, with a number of lineage-specific innovations pointing to tunicates as a particularly interesting group for comparative genomic studies [4]. Meanwhile, recent phylogenomic analyses have refuted the classical view by demonstrating that tunicates, not cephalochordates, are the closest living relatives of vertebrates [5-9].

Despite their key position in the tree of life, our comprehension of the phylogenetic affinities within the tunicates is still limited. Traditionally, tunicates have been classified into three major classes – Ascidiacea, Appendicularia and Thaliacea – with distinct life-history traits and developmental modes. Among these classes, the Ascidiacea, commonly referred to as ascidians, is by far the most diversified group, with more than 2500 species classified on the basis of the structure of their branchial sac into three orders: Phlebobranchia, Aplousobranchia and Stolidobranchia [10-12]. Ascidians begin their lives as tadpole-like swimming larvae that later undergo a metamorphosis resulting in morphologically modified, sac-like sessile adults that can be either solitary or colonial. In contrast, the Thaliacea and Appendicularia classes consist of exclusively planktonic species. Appendicularia are peculiar in departing from the common developmental program of metamorphosis. They retain larval characteristics for their entire lifespan and are consequently of pivotal interest from an evolutionary developmental point of view [13].

Over the last decade, molecular phylogenetic analyses have been introduced to shed light on tunicate relationships. Following the pioneering study of Wada [14], the 18S rRNA gene had been the main marker used in reconstructing tunicate relationships at different taxonomic scales [15-19]. In all these studies, molecular phylogenies have contradicted the traditional classification, especially when higher taxonomic levels are considered. Molecular data refuted the division into the three classes (Ascidiacea, Thaliacea and Appendicularia), arguing instead for more complex evolutionary relationships among members of these morphologically distinct groups [20]. In overview, 18S rRNA data have clearly supported the paraphyletic nature of the class Ascidiacea by dividing the tunicates into the following three clades: Phlebobranchia + Thaliacea, Appendicularia, and Stolidobranchia, the last comprising Molgulidae and Pyuridae + Styelidae [15-19]. However, while this scheme is generally accepted, several major phylogenetic questions remain unanswered, among which at least three are prominent.

The first involves the long-standing question of the position of Appendicularia, which is crucial for understanding the evolution of body plans and developmental modes in tunicates [13]. One problem with positioning appendicularians in tunicate phylogeny is that the two 18S rRNA sequences from the genus *Oikopleura*, the only representatives currently sampled, evolved at an elevated rate [14]. This high evolutionary rate seems to be a genome-wide characteristic in *Oikopleura dioica *and leads to problematic long branches in inferred molecular phylogenies [6]. Consequently, in the majority of phylogenetic studies conducted so far, Appendicularia occupy a basal position in being the sister-group of all other tunicates [14,17,21]. This basal position, which might reflect a long-branch attraction artefact [17], has recently been challenged by analyses, including an improved taxon sampling, that instead favoured a sister-group relationship of Appendicularia with Stolidobranchia [19,20].

The second irresolution concerns the placement of the ascidian order Aplousobranchia within the tunicates. All aplousobranch species are colonial and constituted by small zooids often embedded in a common tunic. In early 18S rRNA studies, it was proposed that Aplousobranchia form the sister-group of Appendicularia and that together they occupy the most basal position among tunicates [16]. Later on, phylogenies based on analyses of the mitochondrial cytochrome oxidase subunit I gene (*cox1*) suggested a close affinity of Aplousobranchia with Cionidae and Diazonidae (Phlebobranchia) [22], which is also supported by morphological characters [23]. Recently, it was shown that the spurious relationship of Aplousobranchia with Appendicularia [16] was an artefact due to the contamination of aplousobranch 18S rRNA sequences by protistan symbionts [18], and analyses of authentic 18S rRNA sequences clustered Aplousobranchia with Thaliacea [18]. However, the 18S rRNA sequences of Aplousobranchia seemed to evolve at extraordinarily high rates, yielding extremely long branches in the inferred phylogenetic trees [18]. This renders it particularity difficult to obtain a reliable placement of Aplousobranchia within the tunicates [19,20].

The third uncertainty pertains to the phylogenetic relationships of the thaliaceans within the ascidians. Despite the fact that rRNA studies have always placed Thaliacea and Phlebobranchia together [17-20], the exact phylogenetic position of Thaliacea remains unclear. This is mainly due to poor taxonomic sampling, with only three representatives of Thaliacea included to date in 18S rRNA phylogenies. The high evolutionary rate of 18S rRNA sequences in Thaliacea also precluded firmly resolving the phylogenetic relationships among its three constitutive orders.

Most of our knowledge of tunicate molecular phylogeny comes from analyses of 18S rRNA sequences using standard models of nucleotide sequence evolution [18,19]. Although among site-rate heterogeneity can be efficiently modelled by fitting a gamma-distribution [24] or by assuming a proportion of invariable sites [25], these standard models make several assumptions, such as the independent evolution of nucleotide sites, and the spatial homogeneity of the substitution process across sites. In this respect, it has been demonstrated that neglecting the co-evolving paired sites in stems affects the estimation of bootstrap values [26,27] and also influences topological inference [28-30]. The spatial substitution pattern heterogeneity of sequence evolution may also produce misleading phylogenetic signals [31,32] which can severely affect phylogenetic reconstruction in cases of high substitutional saturation [33]. Our aim was thus to establish an updated phylogenetic framework for tunicate evolution based on analyses of the 18S rRNA gene, using the most realistic models that account for secondary structure and across-site heterogeneities in the evolutionary process. Phylogenetic analyses of a dataset incorporating 30 new complete 18S rRNA tunicate sequences allowed us to compare for the first time the use of RNA-specific paired-site substitution models [29,30,34] and the CAT mixture model [31], relaxing the hypothesis of a uniform substitution process by letting patterns of substitution be distinct at different sites.

## Results and discussion

### Improved phylogenetic models and 18S rRNA evolution

Table 1 summarises the selected models and methods used for phylogenetic inference of the two assembled datasets: a 110-taxon dataset including all tunicate sequences and a reduced 88-taxon dataset excluding the highly divergent aplousobranch species (see Material and Methods). The tree-reconstruction approaches we followed can be divided into three categories based on the type of substitution models used: (1) standard independent-site DNA models (e. g. GTR+Γ +I); (2) doublet or paired-site substitution models (e. g. RNA6A+Γ+I); and (3) the CAT-GTR non-parametric mixture model.

**Table 1:** Phylogenetic approaches and best-fitting models.

Datasets	Phylogenetic Reconstruction Approaches				
	Maximum Likelihood	Bayesian Inference			
	**Standard DNA model**	**Standard DNA model**	**Doublet model**		**Mixture model**

			*Loops:*	*Stems:*	
Complete dataset	GTR + Γ + I	GTR + Γ + I	GTR + Γ + I	RNA6C + Γ + I	CAT-GTR+Γ
110 taxa				RNA7B + Γ	
1326 sites				RNA16A + Γ + I	
					
			*Loops:*	*Stems:*	
Reduced dataset	TN93 + Γ + I	GTR + Γ + I	TN93 + Γ + I	RNA6CA + Γ + I	CAT-GTR+Γ
88 taxa				RNA7B + Γ	
1650 sites				RNA16A + Γ	

Contrary to standard models which assume that all sites evolve independently, doublet or paired-site models include a wide range of substitution models developed to overcome this assumption in rRNA molecules [35]. Ribosomal RNAs are a mosaic of two main structural motifs: single-stranded domains (loops) and double-stranded helices (stems) formed by Watson-Crick base pairing between nucleotides. During rRNA evolution, compensatory substitutions occur regularly in stems in order to maintain the counterpart-paired nucleotides. Doublet models, by considering pairs of nucleotides as states in the substitution matrices, account for this dependence.

There are three classes of doublet models – 6, 7 and 16 state models – differing in their treatment of non-Watson-Crick pairs (mismatches) [35]. For determining the most adequate model, we first selected the best-fitting standard DNA model for the unpaired loop partition, which we subsequently kept fixed in likelihood estimations, varying only the model for the stem partition [see Additional file 1]. For each of the three classes of doublet models, we used the AIC criterion to determine the best-fitting substitution model (i.e., the number of parameters of the transition matrix). However, we could not test whether the 6, 7 or 16 state models were the most appropriate because there is currently little agreement on whether it is possible to compare models with different matrix dimensions. Trees obtained under the best fitting model were almost identical among the three model classes (data not shown). We therefore only discuss those reconstructed using 6-state RNA models, which offer the best compromise between the increase in likelihood and the lowest number of free parameters incorporated.

In the third reconstruction approach, we used a mixture-model analysis to relax the assumption of a homogeneous evolutionary process across sites of the 18S rRNA molecule. The non-parametric mixture model CAT-GTR [31] was chosen for two reasons. First, previous studies using the CAT mixture model showed that it handles the substitutional saturation of the data better than classical homogeneous models [31], hence alleviating phylogenetic artefacts [33]; and second, because in contrast to other mixture models [32], it does not specify the number of mixture components *a priori*. More precisely, the CAT-GTR model assumes an infinite mixture (based on a Dirichlet process) of GTR matrices that differ only in their equilibrium base frequencies. Thus, the CAT model assigns to each site a frequency vector or so called "profile", with the number of different profiles (*K*) being a free parameter.

In statistical analyses, the selection of the model that best fits the data is a prerequisite. Currently, there is no generally accepted way to evaluate with confidence which of the afore-mentioned strategies (standard homogenous model, doublet models or CAT model) offers the best statistical fit for a given dataset. An adequate evaluation would require calculating the Bayes factor between each pair of models using thermodynamic integration [36], but such a strategy is not currently affordable computationally. Nevertheless, several studies have shown that doublet models outperform standard DNA models in phylogenetic reconstructions based on rRNA data [28,29,34,37].

**Figure 1:**
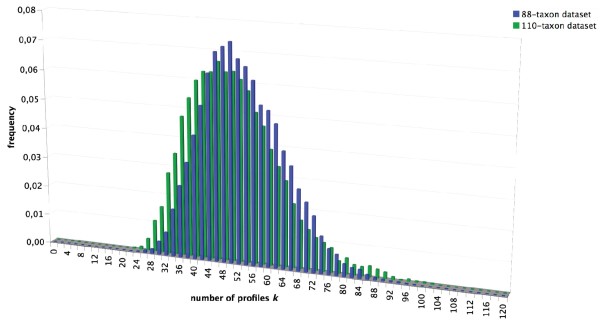
**Estimated number of profiles *K *under the CAT-GTR+Γ mixture model**. The frequencies of the value (numbers of different profiles *K*), as estimated through the MCMC runs at the stationary stage for both the 110-taxa (green) and the 88-taxa (blue) datasets.

With regards to the CAT-GTR mixture model versus standard site-homogeneous models, however, evaluations can be made directly from the posterior distribution of the number of profiles (*K*). If there is no heterogeneity across sites in the data, the CAT model will preferentially assign one single frequency vector for all sites [31]. In fact, the standard GTR model is nested within the CAT-GTR model, so that when the number of profiles is equal to one (*K *= 1), all sites evolve under a homogeneous GTR model. The distribution of the number of inferred profiles (*K*) for our two datasets shows that the frequency of the *K *= 1 class is zero in both cases (Figure 1). This implies that the standard GTR model has not been visited through the MCMC runs for either dataset. This means by extension that the site-heterogeneous CAT-GTR mixture model is statistically better at explaining the data than the standard homogeneous GTR model. Furthermore, the minimum number of predicted profiles is always higher than 20 (Figure 1), which suggests that 18S rRNA sequences are subject to more complex evolutionary pressures than those implied by the simple stem/loop partition.

This observation seems biologically relevant considering that beyond the pairing of nucleotides in stems, the rRNA spatial conformation is also affected by the base composition outside of these regions [38]. In this context, the maintenance of other structural motifs (T-loops, hook turns, GNRA tetraloops, bulged-G motifs, k-turns and A-minor motifs) is likely to be selectively constrained. Indeed, these motifs play an essential role in the enzymatic activity of rRNA and in its interaction with ribosomal proteins. Obviously, even if doublet models allow us to capture a major part of the constraints acting on rRNA, they are still far from modelling the overall evolutionary complexity of these molecules.

### Evolutionary shifts in 18S rRNA sequences and phylogenetic relationships of Aplousobranchia

**Figure 2:**
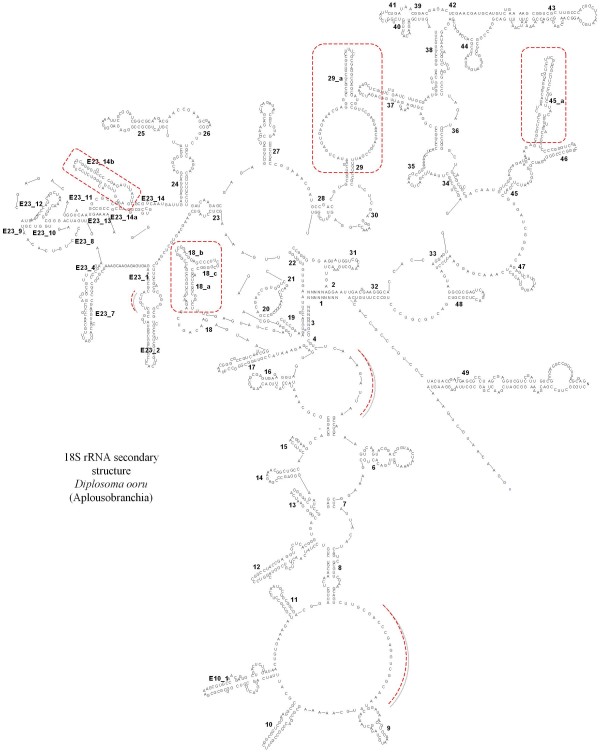
**Predicted 18S rRNA secondary structure of a divergent aplousobranch sequence (*Diplosoma ooru*)**. New predicted structures unique to such divergent Aplousobranchia species (and absent in the conserved *Pycnoclavella *aff. *detorta *and *Clavelina meridionalis *sequences) are boxed in red. Red dotted lines indicate additional loop regions where major elongations occurred in other divergent aplousobranchs.

Most 18S rRNA sequences from species belonging to the divergent order Aplousobranchia are about 300 nucleotides longer than the other typical tunicate ones [18]. That is, sequences belonging to Didemnidae, Polyclinidae, and Polycitoridae, including the newly obtained *Cystodytes *sp., possess large insertions in multiple parts of the molecule when compared to the remaining tunicate and outgroup species. These insertions take the form of elongations of already existing loops. More specifically, they occur in the unpaired helical regions between stems 4 and 5, and between stems 8 and 9, as well as within stems 18a-c, 29, 45a, E23_14b (Figure 2). Some of these insertions also induce changes to the secondary structure of 18S rRNA. Despite their high degree of divergence in terms of evolutionary rate and secondary structure, these unusual aplousobranch sequences are likely to be functional. Indeed, new helical structures are predicted in the elongated regions corresponding to helices 18, E23_14, 29 and 45 of the 18S rRNA molecule (Figure 2). Similar observations have also recently been reported in haplosclerid sponges [30] showing that a certain degree of freedom exists relative to the consensus 18S rRNA structure model. Knowing exactly which families belong to Aplousobranchia has been a matter of debate [23,39]. Here, we obtained sequences from two species belonging to the previously unsampled aplousobranch family Clavelinidae *sensu *Perez-Portela *et al. *[40] (*Clavelina meridionalis *and *Pycnoclavella *aff. *detorta*). Interestingly, these two sequences lack all the Aplousobranchia-specific structures described above, and thus show 18S rRNA sequence lengths more similar to other tunicate families.

**Figure 3:**
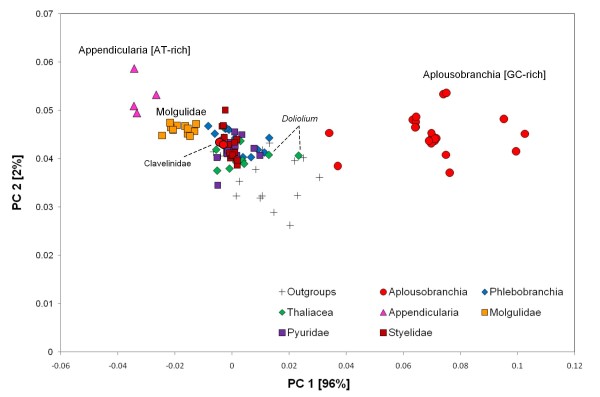
**Analysis of base-composition heterogeneity**. Principal component analysis (PCA) of the base composition of 18S rRNA from the 110-taxa dataset considering all nucleotide sites. The graph shows the first two principal components (PC), which contribute 96% and 2% of the total variance, respectively. The main component represents the variance along the AT versus GC axis, with the AT-rich Appendicularia, and the GC-rich Aplousobranchia at the two extremes.

Beside the greater gene length and the modified secondary structure, a clear compositional bias was detected for the Aplousobranchia by a Principal Component Analysis (PCA) of base composition (Figure 3). The PCA revealed that the 18S rRNA sequences of the non-clavelinid Aplousobranchia have a different base composition than other tunicates and the outgroup species. That is, the sequences of Didemnidae, Polycitoridae and Polyclinidae are GC-rich. Conversely, Appendicularia and Molgulidae, are the most AT-rich according to the PCA results (Figure 3). The exact same compositional differences were evidenced when the loop and stem regions of the sequences were analysed separately (data not shown). Once again, the Clavelinidae sequences differ from those of other Aplousobranchia and have a base composition like that of typical tunicates and the outgroups (Figure 3).

**Figure 4:**
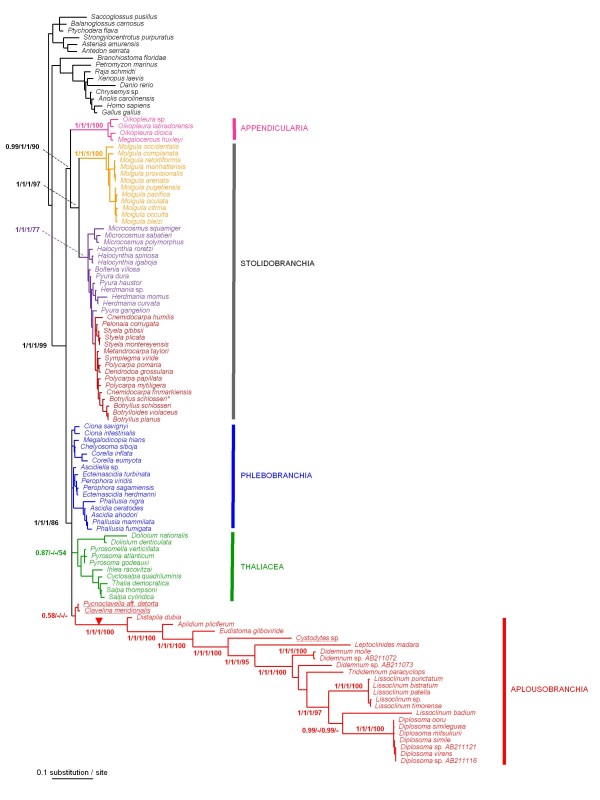
**Phylogeny of tunicates inferred from the complete 18S rRNA dataset (110 taxa and 1373 sites)**. Bayesian majority-rule consensus tree obtained under the CAT-GTR+Γ mixture model implemented in PhyloBayes. Support values at nodes represent: Bayesian Posterior Probabilities (PP) obtained under: 1. PP1 = CAT-GTR+Γ (PhyloBayes)/2. PP2 = RNA6C+Γ+I and GTR+Γ+I (Phase)/3. PP3 = GTR+Γ+I (MrBayes)/4. BP = Maximum likelihood bootstrap percentages (BP) under GTR+Γ+I (PAUP*). Support values are indicated for the main tunicate clades, and within Aplousobranchia, when PP ≥ 0.95 and BP ≥ 65. Newly sequenced Aplousobranchia species are underlined. Among the Stolidobranchia, a newly obtained sequence from *Botryllus schlosseri *is marked with an asterisk. The red triangle indicates the evolutionary shift in secondary structure of the 18S rRNA molecule within Aplousobranchia.

The phylogenetic analysis of the complete tunicate dataset (110 taxa, including 95 tunicate species, and 1326 unambiguously aligned nucleotide sites: Table 1) revealed particularly long branches for the three Aplousobranchia families Polyclinidae, Polycitoridae and Didemnidae (Figure 4), as reported previously [18]. This dramatic rate acceleration was found regardless of the probabilistic method and the evolutionary model used. Furthermore, the newly obtained sequence from *Cystodytes sp. *(Polycitoridae) also had an elevated evolutionary rate and fell squarely within Aplousobranchia with high support. In striking contrast, the two sequences of the Clavelinidae evolved remarkably slowly compared to other Aplousobranchia species (Figure 4).

From the phylogenetic point of view, the two slowly evolving Clavelinidae grouped together as a sister-group to the three divergent Aplousobranchia families in Bayesian analyses under the CAT mixture model and in Maximum Likelihood (ML) analyses, but with a lack of nodal support (Figure 4). This result is in agreement with what was recently proposed on the basis of the mitochondrial gene *cox1 *[22]. However, monophyly of Aplousobranchia, including Clavelinidae, was not obtained in Bayesian analyses under secondary-structure models or site-homogeneous DNA models. These models instead yielded a tree topology where the divergent aplousobranch families (Polyclinidae, Polycitoridae and Didemnidae) clustered within Thaliacea to the exclusion of the Clavelinidae species. More precisely, a sister-group relationship to species of the genus *Doliolum *was obtained, but also with poor support values (data not shown).

**Figure 5:**
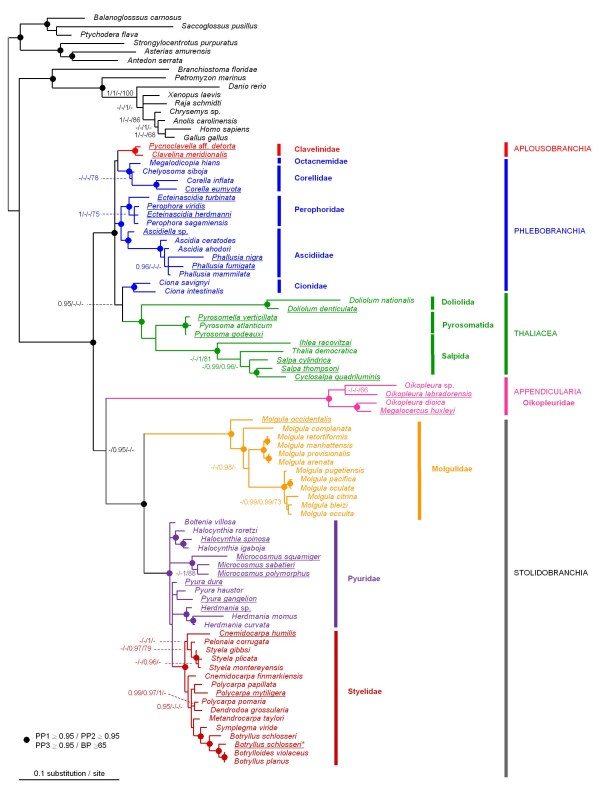
**Phylogeny of tunicates inferred from a reduced 18S rRNA dataset (88 taxa and 1675 sites)**. Bayesian majority-rule consensus tree obtained under the CAT-GTR+Γ mixture model implemented in PhyloBayes after exclusion of the fast-evolving Aplousobranchia species. Support values obtained using different reconstruction approaches are indicated at nodes in the following order: Bayesian posterior probabilities (PP) under: 1. PP1 = CAT-GTR+Γ (PhyloBayes)/2. PP2 = RNA6A+Γ+I and TN93+Γ+I (Phase)/3. PP3 = GTR+Γ+I (MrBayes)/and 4. BP = Maximum Likelihood bootstrap percentages (BP) under TN93+Γ+I (PAUP*). Support values are displayed when PP ≥ 0.95 and BP ≥ 65. Dots indicate nodes for which all four reconstruction methods agree and provide PP ≥ 0.95 and BP ≥ 65. Newly obtained sequences are underlined, including an additional one from *Botryllus schlosseri *marked with an asterisk.

Several arguments suggest that the previously proposed branching of Aplousobranchia within Thaliacea [18] might be a long-branch attraction (LBA) artefact [41] combined with a compositional effect. First, the topology is unstable and this grouping is not longer supported by the clavelinid sequences when the divergent aplousobranch species are removed (see Figure 5). Also, when the *Doliolum *species were removed from the analyses, the Aplousobranchia affinity with the remaining Thaliacea was not recovered (data not shown). Second, Doliolida is the second fastest-evolving group in the Phlebobranchia + Thaliacea + Aplousobranchia clade and this could promote LBA to the fastest evolving Aplousobranchia. Third, the PCA of base composition revealed that the slightly GC-rich sequences from the *Doliolum *species are the closest in composition among tunicates to the deviant aplousobranch sequences (Figure 2), another potential cause of the observed phylogenetic artefact.

In conclusion, our results are consistent with the traditional view that the aplousobranch families Clavelinidae, Didemnidae, Polycitoridae and Polyclinidae form a monophyletic group which implies the occurrence within this order of drastic shifts in secondary structure, base composition, and evolutionary rate. Our phylogenetic analyses also provide firm support for their clustering with Phlebobranchia and Thaliacea species in a common clade. On the other hand, because of the peculiarities of 18S rRNA evolution in most species of Aplousobranchia, this gene does not allow establishing the phylogenetic relationships of this order with certitude. Also, the taxonomic sampling of 18S rRNA sequences is still insufficient within Aplousobranchia, because the diversity of this group is huge and may comprise more than the five families here included [23,42]. Further work is needed for gaining an accurate picture of Aplousobranchia evolution within tunicates.

### An updated 18S rRNA phylogenetic picture for tunicates

The growing interest in tunicates, along with the realisation that they are the sister-group of vertebrates [5-9], reinforces the importance of reconstructing a reliable phylogenetic framework for their evolutionary history. Previous molecular studies have revealed that tunicate phylogenetic relationships are difficult to resolve, mainly because of marked differences in evolutionary rates among lineages [16-19,43]. Among others, Appendicularia, Aplousobranchia, and Thaliacea have been the typical examples of rapidly evolving groups. In this respect, we aimed at revisiting tunicate 18S-rRNA phylogeny by using a wider taxonomic sampling targeted at these crucial groups, and by applying a range of phylogenetic-reconstruction approaches in a probabilistic framework. Under this general scheme, we re-evaluated previously proposed relationships and the resulting evolutionary scenarios.

#### I. Ascidiacea paraphyly and tunicate systematics

The different reconstruction methods applied to the complete dataset unambiguously supported the division of tunicates into the following groups: Phlebobranchia + Thaliacea + Aplousobranchia, Appendicularia and Stolidobranchia (Figure 4). Bayesian analyses provided maximal branch support (PP = 1.0) for the respective monophyly of each group, and good ML bootstrap support was obtained for the monophyly of the clade Phlebobranchia + Thaliacea + Aplousobranchia (BP = 86). A monophyletic Stolidobranchia is also retrieved with strong branch support values grouping monophyletic Molgulidae with Pyuridae + Styelidae (Figure 4). Interestingly, this dataset strongly favoured Appendicularia as the sister-group of Stolidobranchia (BP = 90) (Figure 4).

However, the inclusion of the divergent aplousobranch sequences in this 110-taxon analysis precludes a reliable examination of the phylogenetic relationships among the slower-evolving taxa. In order to obtain a reliable picture of tunicate relationships, the divergent aplousobranch sequences were removed from subsequent phylogenetic analyses, keeping only the slowly evolving Clavelinidae as representatives. This resulted in a dataset with 88 taxa, including 73 tunicates, but encompassing an expanded set of 1650 unambiguously aligned sites (Table 1). Probabilistic analyses of this dataset under standard DNA models using ML or a Bayesian approach yielded congruent results. Comparing these results to the ones obtained in Bayesian analyses using mixture and partitioned-doublet models, some topological differences were observed (compare the different support values in Figure 5).

Concerning the relationships among the major tunicate lineages, all probabilistic analyses of this reduced dataset yielded a phylogenetic pattern similar to that for the complete data set (compare Figure 5 with Figure 4). The two ascidian orders Aplousobranchia and Phlebobranchia again grouped strongly with Thaliacea. Appendicularia was unambiguously monophyletic, as were also Molgulidae, and the grouping of Pyuridae with Styelidae. The monophyly of Stolidobranchia was also strongly recovered (Figure 5). However, in contrast with analyses of the complete dataset, the support for Appendicularia as the sister-group of Stolidobranchia was decreased (Figure 5). Indeed, only the partitioned Bayesian analysis using RNA secondary-structure models provided some support for this relationship (PP = 0.95).

Our results therefore confirm that Ascidiacea, as currently defined, is a paraphyletic group that includes Thaliacea and, most likely, Appendicularia, as previously suggested [14,16-20]. Traditionally, ascidians were subdivided morphologically in two different ways (or combinations thereof): 1) based on the structure of the branchial sac, dividing ascidians into Aplousobranchia, Phlebobranchia and Stolidobranchia, as established by Lahille [10-12]; and 2) based on the position of the gonads, which separates ascidians into Enterogona and Pleurogona, following Perrier [44] as modified by Garstang [45]. Enterogona includes the Aplousobranchia and Phlebobranchia, while Pleurogona consists of Stolidobranchia (Figures 4 and 5). The finding that 18S rRNA joins Phlebobranchia and Aplousobranchia into a common clade supports the classification scheme of Enterogona and Pleurogona. Yet, accepting this view, Thaliacea branched in our trees within Enterogona, and Appendicularia with Pleurogona. The gonad position in the thaliaceans indeed conforms to the enterogonid type (associated with the gut), while in the appendicularians the highly modified metamorphosis renders any inference about the gonad position difficult.

#### II. Phlebobranchia and the sister-group Cionidae

Within Phlebobranchia, the five represented families – Ascidiidae, Perophoridae, Octacnemidae, Corellidae and Cionidae – appeared reciprocally monophyletic with high support values, except for Corellidae (Figure 5). At the inter-familial level, strong support was obtained for grouping Octanemidae with Corellidae, and Perophoridae with Ascidiidae within which *Ascidiella *appears divergent from the closely related genera *Phallusia *and *Ascidia *(Figure 5). The inclusion of Cionidae within Aplousobranchia instead of in its traditional position within Phlebobranchia has been suggested on the basis of both morphological characters [23] and analyses of *cox1 *[22]. However, this grouping was not recovered in any of our trees, which are consistent with the traditional phlebobranch position of Cionidae. Our results support the paraphyly of Phlebobranchia, with a close phylogenetic affinity between the *Ciona *species and Thaliacea in Bayesian analyses using the CAT-GTR mixture model (PP = 0.95). Although not supported by more conventional reconstruction methods (Figure 5), such a relationship provides a good working hypothesis for future phylogenomic studies aiming at determining the sister-group of model species from the *Ciona *genus.

#### III. Thaliacea relationships

The Thaliacea are a class of exclusively planktonic tunicates and consist of three main orders: Doliolida, Pyrosomatida and Salpida. They are distinct from the other tunicates, because all members of this class form specialized colonies and have complex life cycles, characterized by the alternation between sexual and asexual generations. There were two traditional views on the origin of the class: the first suggesting that Thaliacea derived from an Appendicularia-like ancestor (Herdman 1888; Seeliger 1893 – 1911; Newman 1933 cited in [46]), and the second deriving Thaliacea from an ascidian-like ancestor [47-49] or even placing the split more basally, with the ascidians and thaliaceans evolving from a common ancestor [45]. In parallel to this controversy, there has been considerable debate on whether the thaliaceans are monophyletic or not [46]. Within Thaliacea, however, the commonly accepted view places the pyrosomatids in a basal position, with the doliolids and salps being sister-groups, following a trend toward branchial sac simplification and muscle-band development [47].

Meanwhile, early molecular phylogenies supported a close relationship between Thaliacea and Phlebobranchia thereby suggesting Thaliacea arose from a sessile ascidian-like ancestor [14]. Then, Thaliacea was also reported to be monophyletic in more recent studies [16,19,20]. On the phylogenetic affinities among its three constitutive orders, the phylogenetic evidence appears more ambiguous. In fact, in half of the previous 18S-rRNA tunicate phylogenies, Doliolida were found to be the sister-group of Salpida [14,19], whereas in others a closer relationship was recovered between Pyrosomatida and Salpida to the exclusion of Doliolida [16,17].

All previous sampling of Thaliacea was limited to the species *Pyrosoma atlanticum*, *Doliolum nationalis*, and *Thalia democratica*, among which the latter two were characterized by relatively long branches. This evolutionary-rate heterogeneity is probably the reason for the reported conflicting relationships among the three orders. In our study, we subdivided these long branches by tripling the taxonomic sampling which rendered the strong statistical support for the clustering of Thaliacea with Phlebobranchia and Aplousobranchia even more reliable (Figure 5). Although we still lack some representative families, the monophyletic nature of Thaliacea and the respective monophyly of its three constitutive orders were also firmly supported in all reconstructed trees (Figure 5). This is in agreement with previous taxonomic views (e.g., [47,48]).

Regarding thaliacean interrelationships, our phylogeny suggests a sister-group relationship between Pyrosomatida and Salpida, with Doliolida species arising first (Figure 5). Such a relationships was obtained in Bayesian analyses using mixture (PP = 0.69) and secondary structure (PP = 0.56) models whereas analyses conducted under standard DNA models (PP = 0.69; BP = 57) tend to group the two fast-evolving groups Salpida and Doliolida as reported in the most recent study [19]. These contrasting results suggested a possible effect of model misspecification leading to a potential LBA artefact grouping the fast evolving Salpida and Doliolida within Thaliacea in analyses conducted under standard DNA models. If confirmed, this particular case probably represents an example where the use of the most complex and best fitting models of sequence evolution allows improving phylogenetic inference [29,33].

Our proposed phylogenetic hypothesis seems at first counterintuitive since both doliolids and salps have solitary generations, in contrast to pyrosomes, which are exclusively colonial and are generally considered as the least specialized thaliaceans. However, the notion of a basal Pyrosomatida from which Doliolida and Salpida derive is at odds with the fact that only Doliolida in this group retain the larval features of tail and notochord. In contrast, our phylogenetic picture implies a single event of tail loss within the thaliacean lineage. In agreement with this view of a basal Doliolida, comparative studies of sperm morphology have shown that doliolid sperm is more plesiomorphic than that of the pyrosomes or salps [46,50]. Likewise, the simple arrangement of gill slits and the particular morphogenesis and structure of stolons in pyrosomes and salps may represent synapomorphic characters of the two groups [51].

#### IV. The phylogenetic position of Appendicularia

Appendicularians or Larvaceans are small planktonic tunicates that have short generation times and characteristic tadpole body plans, consisting of a short trunk and a posterior tail with a notochord and a dorsal neural tube. From a developmental point of view they are unique among tunicates, since all species belonging to this class retain the tail and the notochord in adulthood [52]. For this reason, the relationships of Appendicularia to the other tunicate lineages have always been considered as crucial for understanding the evolution of chordate body plans [14,16]. The traditional classification distinguishes three families within the class Appendicularia: Fritillariidae, Kowalevskiidae and Oikopleuridae [53]. However, all previous molecular phylogenies have been taxonomically restrained to species from the model genus *Oikopleura *(Oikopleuridae). While early studies of 18S rRNA indicated that Appendicularia emerged first among tunicates [14,17], more recent analyses suggested a sister-group relationship with Stolidobranchia [19]. Their phylogenetic position remains controversial mainly because *Oikopleura *18S rRNA sequences are highly evolving [17,43].

Our results, including two additional species belonging to Oikopleuridae (*Oikopleura labradorensis *and *Megalocercus huxleyi*), did not seem to break up the long appendicularian ancestral branch enough to firmly establish the position of this order within tunicates. Indeed, whereas the complete dataset strongly favoured Appendicularia as the sister-group of Stolidobranchia (Figure 4), in the reduced but less heterogeneous dataset (Figure 5), only the partitioned Bayesian analysis using RNA-secondary structure models provided support for this relationship (PP = 0.95). These results do not support the basal emergence of Appendicularia [14,16,17], suggesting instead their placement as a sister-group to Stolidobranchia, as recently proposed [19]. The most parsimonious interpretation of this internal position would imply that the planktonic Appendicularia derived from a sessile ascidian-like ancestor.

Still, a reliable placement for Appendicularia based on 18S rRNA data is hampered by two potentially confounding factors: the persisting long branches characterizing the group, and the similarity of nucleotide composition in Appendicularia and Molgulidae (Figure 2). In both, 18S rRNA sequences are AT-rich and experienced accelerated evolutionary rates, leaving open the possibility of an artefactual attraction of the two groups due to systematic biases. Finally, phylogenomic studies, although based on just a few taxa, strongly support the basal position of Appendicularia within tunicates [6,7]. However, *Oikopleura dioica*, the sole member of the class with currently available genomic data, evolves at such an elevated rate that any conclusion concerning its exact phylogenetic position based on these data would be premature.

#### V. Stolidobranchia

Stolidobranchia appears as unambiguously monophyletic in all phylogenetic reconstructions performed (Figures 4 and 5). Within Stolidobranchia, Molgulidae formed a monophyletic group characterized by accelerated evolutionary rates compare to other stolidobranchs (Figure 5). The basal emergence of *Molgula occidentalis *was significantly supported (0.97/1.0/1.0/88), followed by *Molgula complanata *as the sister-group of two reciprocally monophyletic groups that include other molgulids, in good agreement with a previous study of the group based on partial sequences [15].

Overall, phylogenetic affinities within Molgulidae were rather well resolved and stable, whereas Pyuridae, and especially, Styelidae showed greater topological instability (Figure 5). Despite the addition of 10 species belonging to both Pyuridae and Styelidae, phylogenetic relationships among and within the two families were crippled by low resolution, with two multifurcations occurring within Pyuridae and poor support values at several nodes within this clade (Figure 5). In our trees, the family Styelidae is monophyletic whereas Pyuridae appears paraphyletic (Figure 5). These results contrast with those from a recent study of the stolidobranchs based on partial 18S rRNA and *cox1 *sequences where the reverse situation was observed [54]. This incongruence might stand in a rooting problem of the Pyuridae-Styelidae group whereby its fast-evolving, and distant molgulid sister-group, created LBA effects [54]. In fact, only the relationships within Botryllinae were well-supported by all reconstruction methods. Furthermore, the inclusion of Botryllinae within Styelidae was strongly supported, upholding the view that the latter is in fact a subfamily of Styelidae [47,55]. Yet, little evidence is provided for the exact position of the Botryllinae, because its proposed sister-group relation to the colonial *Metandrocarpa taylori *[19] was not significantly supported (Figure 5).

## Conclusion

Our work has improved tunicate phylogeny based on 18S rRNA, in order to provide a reliable phylogenetic framework for the evolution of this key group of chordates. This was accomplished by a wider taxonomic sampling than in previous studies, yielding a more thorough representation of major tunicate lineages, and subdividing previously long branches. Our results showed that the non-parametric CAT-GTR mixture model has a better fit than standard DNA models on tunicate 18S rRNA data. The high number of site profiles inferred under the CAT-GTR model also suggested that the *a priori *partition of 18S rRNA data into stem and loop regions is likely an oversimplification of the complex evolutionary constraints acting on tunicate 18S rRNA sequences.

The 18S rRNA gene provided a clear view of the evolution of major tunicate lineages, but appeared less informative for relationships at lower taxonomic levels. This was reflected by the weak support values accorded to several nodes in the reconstructed trees, and by the unstable position of certain groups between phylogenies inferred using different reconstruction approaches. Among the spots of low resolution, the relationships at inter- and intra-familial levels within Phlebobranchia and within the Pyuridae – Styelidae group proved difficult to clarify. The development of new phylogenetic markers is thus necessary for the further comprehension of tunicate evolutionary history. An obvious candidate is the 28S rRNA gene, which is already available for some species and whose resolving power in combination with the 18S rRNA has been widely demonstrated [43,56]. Finally, the increasing availability of genomic information for tunicates also constitutes a promising source of future nuclear protein-coding markers.

## Methods

### Taxon sampling

The tissue samples used in this study were collected from the following species: class Ascidiacea: Phlebobranchia: *Perophora viridis*, *Ecteinascidia herdmanni*, *Ecteinascidia turbinata*, *Ascidiella *sp., *Corella eumyota*, *Phallusia nigra*, *Phallusia fumigata*; Aplousobranchia: *Pycnoclavella *aff. *detorta*, *Clavelina meridionalis*, *Cystodytes *sp.; Stolidobranchia: *Molgula occidentalis*, *Halocynthia spinosa*, *Pyura dura*, *Pyura gangelion*, *Herdmania *sp., *Microcosmus squamiger*, *Microcosmus sabatieri*, *Microcosmus polymorphus*, *Polycarpa mytiligera*, *Botryllus schlosseri*, *Cnemidocarpa humilis*, class Thaliacea: *Doliolum denticulata*, *Pyrosomella verticillata*, *Pyrosoma godeauxi*, *Ihlea racovitzai*, *Salpa cylindrica*, *Salpa thompsoni*, *Cyclosalpa *sp.; and class Appendicularia: *Oikopleura labradorensis*, *Megalocercus huxleyi*.

### DNA extraction, amplification and sequencing

Genomic DNA was extracted from 95% ethanol-preserved tissue samples using either the QIAamp DNA or the DNeasy Plant Mini kits following the manufacturer's protocols or following the procedure of Bernatzky and Tanksley [57]. The 18S rRNA gene was PCR-amplified either in one fragment (using the primer sets 18S1/18S2 or 18S1/18S_Herdm_R1) or in two overlapping fragments of approximately 1 kb, 18S1/18S4 and 18S3/18S2, using the following primers: 18S1 (Fwd) 5'-CCTGGTTGATCCTGCCAG-3', 18S2 (Rev) 5'-TAATGATCCATCTGCAGG-3', 18S3 (Fwd) 5'-TTAGAGTGTTCAAAGCAGGC-3', 18S4 (Rev) 5'-GATTAAAGAAAACATTCTTGGC-3' and the newly designed 18S_Herdm_R1 (Rev) 5'-GATTRACCCGAGACCGCMATTYGCRTT-3'. PCR products were purified from 1.2% agarose gel using Gel Extraction Kit (Millipore) or using polyethylene glycol (PEG) in saline (NaCl). Most of the products were directly sequenced using Big Dye Terminator v1.1 (Applied Biosystems) on an ABI 310 sequencer. Some PCR products were ligated into pGEM-T Easy vector for cloning into One Shot TOP10 Competent Cells (Invitrogen), and five clones per species were sequenced. All chromatograms were manually corrected and assembled using the software Sequencher. The 30 new sequences have been deposited in the EMBL database under Accession Numbers FM244840 to FM244869.

### Data assembly and alignments

In addition to the 30 sequences obtained for this study, more tunicate 18S rRNA sequences with > 85% length coverage were recovered from GenBank [see Additional file 2]. Fifteen outgroups were chosen to evenly sample the diversity of the other phyla of Deuterostomes, with representative species belonging to Vertebrata and Cephalochordata (Chordata), and Hemichordata and Echinodermata (Ambulacraria). In all analyses, the outgroup was used for rooting trees *a posteriori*. Primary multiple alignments were performed using MAFFT [58] and were subsequently adjusted by eye. Following secondary structure models for the 18S rRNA molecule, available in the European Ribosomal Database [59], two partitions or character groups were assigned to the sequences: (1) paired *stem *– characters forming helices in the secondary structure and (2) unpaired *loop *– characters forming single strands. Further alignment adjustments were made manually and by using the script Xstem [29] in order to ensure that all sites in predicted helices form AU, GC Watson-Crick or GU wobble bonds with their partner nucleotide. Finally, ambiguously aligned sites as well as sites including gaps in more than 50% of the sequences were excluded using Gblocks [60] set to the following parameters: Minimum Number Of Sequences For A Conserved Position = n/2 + 1 (where n = number of taxa), Minimum Number Of Sequences For A Flanking Position ≈ 0.85 × n, Maximum Number Of Contiguous Nonconserved Positions = 8, Minimum Length Of A Block = 2 or 5, Allowed Gap Positions = With Half.

Two final datasets were assembled. The first one maximised taxonomic representation by including the Aplousobranchia sequences obtained by Yokobori *et al. *[18] which yielded a matrix of 110 taxa (95 Tunicates + 15 outgroups) and 1373 nucleotide sites. The inclusion of the divergent Aplousobranchia sequences had hindered the alignment of the 18S rRNA regions corresponding to helices E23_1-E23_1', E23_2-E23_2', E23_7-E23_7' and 49-49'. So, the corresponding sites were removed from subsequent phylogenetic analyses. In the second dataset, in order to reduce potential reconstruction artefacts associated with divergent sequences due to long-branch attraction effects [41], the fast-evolving Aplousobranchia sequences were removed. This second dataset had 88 taxa (73 Tunicates + 15 outgroups) and 1675 nucleotide sites.

### Secondary structure prediction and visualisation

The 18S rRNA consensus secondary structure was obtained by following eukaryotic models available in the European Ribosomal RNA Database [59]. Potential structures for specific aplousobranch insertions were predicted using the RNAfold Web Server [61] by choosing the minimum free energy algorithm and the option to avoid isolated base pairs. The predicted secondary structures were visualised using the program RNAviz [62].

### Phylogenetic analyses

Phylogenetic analyses were conducted using Maximum Likelihood (ML) and Bayesian Inference (BI) approaches. In analyses using standard DNA models of sequence evolution, for the entire dataset as well as for the unpaired *loop *partition, the best fitting model was selected using MODELTEST for ML [63] and MrMODELTEST for BI [64] based on the Akaike Information Criterion (AIC) [65]. The best-fitting RNA-specific model for the paired *stem *partition was selected by calculating the AIC from log-likelihoods values previously estimated using the program Optimizer of the PHASE 2.0 package [66]. The selected models for each dataset and for the two different phylogenetic-reconstruction strategies are shown in Table 1.

All ML analyses were performed using PAUP* 4.0b10 [67] using a three round successive-approximation approach for estimating model parameters [68]. Starting from a neighbour-joining (NJ) tree, model parameters were estimated under the likelihood criterion and further kept fixed for heuristic searches with Tree Bisection Reconnection (TBR) branch swapping. The ML tree topology was then kept fixed and model parameters were re-estimated, with the whole process being repeated twice. Statistical support for the nodes was obtained by Bootstrap resampling with 100 pseudo-replicates generated by the program SeqBoot 3.5 of the PHYLIP package [69]. In all replicates, ML analyses were conducted in parallel on a computing cluster using PAUP* through the same heuristic search approach, with model parameters fixed at values estimated previously. Bootstrap Percentages (BP) were obtained from the 50% majority rule consensus of the 100 reconstructed trees using the program TREEFINDER [70]. In all ML analyses, the 6 Ambulacriaria species were declared as outgroup taxa.

Bayesian analyses were conducted using the programs MRBAYES 3.1.2 [71], mcmcphase from the PHASE package [66] and PHYLOBAYES 2.3 [31]. Although both MRBAYES and mcmcphase permit partitioned analysis using RNA secondary structure models, a more complete list of doublet models is implemented in PHASE. Thus in the present study, MRBAYES was principally used for conducting Bayesian phylogenetic reconstructions under a single DNA model of sequence evolution, while mcmcphase was used when both DNA and RNA secondary structure models were considered. Finally, PHYLOBAYES was used for reconstructions under the mixture model CAT, allowing for a general substitution process (CAT-GTR) [31].

In BI analyses conducted with MRBAYES, two independent runs of four incrementally heated Metropolis-coupled Markov chains Monte Carlo (MCMCMC) were launched and run for 5,000,000 generations. In BI analyses conducted with mcmcphase, a single MCMC was launched for 10,000,000 generations. In both cases, parameters and trees were sampled every 100 generations. In PHYLOBAYES, two independent MCMCs were launched for 16,000 to 20,000 cycles with parameters and trees being sampled every cycle (20,000 cycles correspond to about 1,500,000 generations).

In all BI analyses, priors were set to default values and the convergence of chains was double checked. First, values of the marginal likelihood and independent run discrepancies were monitored through generations. Second, the posterior probabilities of all splits were plotted at 20 cycle increments over a run, using the AWTY system [72] [see Additional file 3]. The burnin value was determined when both indicators entered a stationary phase. This typically involved eliminating about 25% of the run samples. Bayesian Posterior Probabilities (PP) were obtained from the 50% majority-rule consensus of the trees sampled during the stationary phase.

## Authors' contributions

FD, XT and EJPD conceived and initiated the study. XT, RRH, YL and NS collected samples and identified specimens. GT, MKT and TF carried out PCR amplifications and sequencing. EJPD, FD, DH and YL supervised the work in their respective labs. GT and FD performed the phylogenetic analyses and drafted the manuscript. All other authors assisted in revising the manuscript. All authors read and approved the final manuscript.

## Supplementary Material

Additional file 1 **Secondary structure model selection**. The table presents the selection of the best-fitting model of rRNA sequence evolution for the 88-taxon and 110-taxon datasets based on the Akaike Information Criterion (AIC). The best-fitting models within each class (6, 7, and 16 states) of doublet models are bold-faced. Click here for file

Additional file 2 **Species sampling, taxonomy and sequence accession numbers**. The table indicate the taxonomy and the species sampling used in the present study with associated sequence Accession Numbers. The 30 new sequences obtained in this study are indicated with a star (*). Click here for file

Additional file 3 **Monitoring the convergence of MCMC in Bayesian analyses**. The figure illustrates the post-analysis of chain convergence in Bayesian analyses under the CAT-GTR+Γ_4 _model for the 88-taxon dataset, using the AWTY system (Nylander *et al*. 2007). A. Cumulative plot of clade posterior probabilities of the 20 more variable splits over a run of 20,000 cycles (i.e. 1,500,000 MCMC generations) sampled at every cycle. The vertical red line indicates the determined burn-in value of 5,000. B. Comparisons of clade posterior probabilities between the two independent MCMC runs. Click here for file
