# Supplementary material for: An updated 18S rRNA phylogeny of tunicates based on mixture and secondary structure models
Source: BMC Evol Biol. 2009 Aug 5;9:187. doi: 10.1186/1471-2148-9-187 (PMC2739199; doi:10.1186/1471-2148-9-187)
Supplement: Additional file 1 — Secondary structure model selection. The table presents the selection of the best-fitting model of rRNA sequence evolution for the 88-taxon and 110-taxon datasets based on the Akaike Information Criterion (AIC). The best-fitting models within each class (6, 7, and 16 states) of doublet models are bold-faced. [file 1471-2148-9-187-S1.pdf]

						88-taxon dataset						110-taxon dataset			
**STEMS**				**LOOPS**						**LOOPS**					
Doublet model	***# RNA***	***# Γ***	***# I***	***Standard DNA model***	***#***	***lnL***	***k***	**AIC$_i$ = -2*lnL*$_i$ + 2*k***	**Δ$_i$ = AIC$_i$ – min AIC**	***Standard DNA model***	***#***	***lnL***	***k***	**AIC$_i$ = -2 *lnL*$_i$ + 2*k***	**Δ$_i$ = AIC$_i$ – min AIC**
**RNA6A+Γ_8+I**	**19**	**1**	**1**	**TN93+Γ_8+I**	**7**	**-15016.54**	**28**	**30089.09**	**0**	**GTR+Γ_8+I**	10	-13611.96	31	27285.91	11.91
**RNA6C+Γ_8+I**	**4**	**1**	**1**			-15032.09	13	30090.19	1.10			**-13621.00**	**16**	**27274.00**	**0**
RNA6C+Γ_8	4	1	0			-15033.68	12	30091.35	2.27			-13622.85	15	27275.70	1.70
RNA6A+Γ_8	19	1	0			-15018.70	27	30091.40	2.32			-13613.37	30	27286.75	12.75
RNA6B+Γ_8+I	7	1	1			-15031.25	16	30094.50	5.41			-13618.95	19	27275.90	1.89
RNA6B+Γ_8	7	1	0			-15032.93	15	30095.85	6.77			-13620.78	18	27277.55	3.55
RNA6D+Γ_8+I	3	1	1			-15110.97	12	30245.93	156.84			-13705.85	15	27441.71	167.71
RNA6D+Γ_8	3	1	0			-15113.62	11	30249.24	160.15			-13706.28	14	27440.56	166.56
RNA6E+Γ_8+I	6	1	1			-15110.21	15	30250.43	161.34			-13703.79	18	27443.58	169.58
RNA6E+Γ_8	6	1	0			-15112.79	14	30253.57	164.48			-13704.24	17	27442.48	168.48
RNA6A+I	19	0	1			-15201.07	27	30456.14	367.06			-13674.26	30	27408.53	134.52
RNA6C+I	4	0	1			-15225.03	12	30474.05	384.96			-13685.03	15	27400.07	126.07
RNA6B+I	7	0	1			-15222.51	15	30475.03	385.94			-13682.39	18	27400.78	126.77
RNA6E+I	6	0	1			-15411.16	14	30850.32	761.23			-13932.88	17	27899.76	625.76
RNA6D+I	3	0	1			-15415.00	11	30852.01	762.92			-13937.24	14	27902.47	628.47
RNA6A	19	0	0			-15518.87	26	31089.73	1000.65			-13833.80	29	27725.60	451.60
RNA6C	4	0	0			-15543.00	11	31107.99	1018.91			-13844.17	14	27716.34	442.34
RNA6B	7	0	0			-15541.41	14	31110.82	1021.74			-13841.93	17	27717.86	443.86
RNA6D	3	0	0			-15793.19	10	31606.37	1517.28			-14149.13	13	28324.25	1050.25
RNA6E	6	0	0			-15790.20	13	31606.40	1517.32			-14144.50	16	28320.99	1046.99
**RNA7B+Γ_8**	**23**	**1**	**0**	**TN93+Γ_8+I**		**-17211.50**	**31**	**34485.00**	**0**	**GTR+Γ_8+I**		**-15311.78**	**34**	**30691.57**	**0**
RNA7B+Γ_8+I	23	1	1			-17211.40	32	34486.80	1.80			-15311.22	35	30692.43	0.87
RNA7A+Γ_8	26	1	0			-17211.38	34	34490.77	5.77			-15311.24	37	30696.48	4.91
RNA7A+Γ_8+I	26	1	1			-17211.29	35	34492.58	7.58			-15310.70	38	30697.39	5.83
RNA7F+Γ_8	6	1	0			-17233.97	14	34495.94	10.94			-15334.43	17	30702.85	11.29
RNA7F+Γ_8+I	6	1	1			-17233.72	15	34497.43	12.43			-15333.47	18	30702.94	11.38
RNA7D+Γ_8	9	1	0			-17233.18	17	34500.36	15.36			-15331.32	20	30702.64	11.08
RNA7D+Γ_8+I	9	1	1			-17232.89	18	34501.79	16.79			-15330.34	21	30702.68	11.11
RNA7C+Γ_8	15	1	0			-17285.43	23	34616.86	131.86			-15415.84	26	30883.69	192.12
RNA7C+Γ_8+I	15	1	1			-17285.09	24	34618.19	133.19			-15415.71	27	30885.43	193.86
RNA7E+Γ_8	7	1	0			-17312.78	15	34655.55	170.55			-15439.92	18	30915.85	224.28
RNA7E+Γ_8+I	7	1	1			-17311.95	16	34655.90	170.90			-15439.36	19	30916.73	225.16
RNA7B+I	23	0	1			-17495.45	31	35052.91	567.91			-15415.89	34	30899.78	208.21
RNA7A+I	26	0	1			-17495.43	34	35058.85	573.85			-15413.94	37	30901.88	210.32
RNA7D+I	9	0	1			-17519.18	17	35072.36	587.36			-15429.86	20	30899.72	208.15
RNA7F+I	6	0	1			-17522.23	14	35072.46	587.46			-15434.45	17	30902.89	211.33
RNA7C+I	15	0	1			-17638.98	23	35323.96	838.96			-15615.12	26	31282.25	590.68
RNA7E+I	7	0	1			-17657.74	15	35345.47	860.48			-15630.62	18	31297.25	605.68
RNA7B	23	0	0			-17763.13	30	35586.26	1101.26			-15551.30	33	31168.59	477.03
RNA7A	26	0	0			-17762.62	33	35591.23	1106.23			-15549.91	36	31171.82	480.25
RNA7F	6	0	0			-17787.60	13	35601.20	1116.20			-15569.99	16	31171.98	480.41
RNA7D	9	0	0			-17786.13	16	35604.27	1119.27			-15566.67	19	31171.35	479.78
RNA7C	15	0	0			-17900.15	22	35844.30	1359.30			-15751.25	25	31552.49	860.93
RNA7E	7	0	0			-17914.08	14	35856.16	1371.16			-15767.71	17	31569.42	877.86
**RNA16A+Γ_8**	**19**	**1**	**0**	**TN93+Γ_8+I**		**-18387.58**	**27**	**36829.17**	**0**	**GTR+Γ_8+I**		-16256.19	30	32572.39	1.33
**RNA16A+Γ_8+I**	**19**	**1**	**1**			-18387.03	28	36830.05	0.89			**-16254.53**	**31**	**32571.06**	**0**
RNA16C+Γ_8	10	1	0			-18406.76	18	36849.53	20.36			-16266.82	21	32575.64	4.58
RNA16C+Γ_8+I	10	1	1			-18406.27	19	36850.55	21.38			-16265.11	22	32574.21	3.15
RNA16D+Γ_8	6	1	0			-18433.45	14	36894.91	65.74			-16306.94	17	32647.88	76.82
RNA16E+Γ_8	5	1	0			-18522.36	13	37070.71	241.54			-16369.39	16	32770.79	199.73
RNA16F+Γ_8	5	1	0			-18559.27	13	37144.54	315.37			-16391.11	16	32814.21	243.15
RNA16I+Γ_8	20	1	0			-18566.60	28	37189.21	360.04			-16412.63	31	32887.26	316.20
RNA16I+Γ_8+I	20	1	1			-18566.60	29	37191.20	362.03			-16412.01	32	32888.01	316.95
RNA16J+Γ_8	17	1	0			-18572.02	25	37194.03	364.87			-16412.84	28	32881.67	310.61
RNA16J+Γ_8+I	17	1	1			-18571.99	26	37195.97	366.80			-16412.26	29	32882.53	311.46
RNA16K+Γ_8	16	1	0			-18583.29	24	37214.58	385.41			-16418.33	27	32890.67	319.61
RNA16K+Γ_8+I	16	1	1			-18583.11	25	37216.22	387.05			-16417.74	28	32891.48	320.42
RNA16B+Γ_8	15	1	0			-18600.57	23	37247.14	417.97			-16442.31	26	32936.63	365.57
RNA16B+Γ_8+I	15	1	1			-18599.71	24	37247.43	418.26			-20157.95	27	40369.89	7798.83
RNA16A+I	19	0	1			-18701.22	27	37456.45	627.28			-16373.58	30	32807.17	236.11
RNA16C+I	10	0	1			-18722.61	18	37481.21	652.04			-16384.94	21	32811.88	240.82
RNA16A	19	0	0			-18984.52	26	38021.03	1191.86			-16523.25	29	33104.51	533.45
RNA16C	10	0	0			-19006.51	17	38047.01	1217.84			-16533.17	20	33106.34	535.28
RNA16I+I	20	0	1			-19079.33	28	38214.65	1385.48			-16709.99	31	33481.97	910.91
RNA16J+I	17	0	1			-19082.68	25	38215.35	1386.18			-16711.79	28	33479.59	908.53
RNA16K+I	16	0	1			-19089.46	24	38226.93	1397.76			-16712.97	27	33479.93	908.87
RNA16B+I	15	0	1			-19099.62	23	38245.25	1416.08			-16714.15	26	33480.31	909.25
RNA16D	6	0	0			-19189.74	13	38405.49	1576.32			-16726.93	16	33485.86	914.80
RNA16F	5	0	0			-19275.60	12	38575.20	1746.03			-16768.85	15	33567.70	996.64
RNA16E	5	0	0			-19481.25	12	38986.50	2157.33			-16922.75	15	33875.50	1304.44
RNA16I	20	0	0			-19496.02	27	39046.03	2216.86			-16953.11	30	33966.22	1395.16
RNA16J	17	0	0			-19501.33	24	39050.67	2221.50			-16955.99	27	33965.98	1394.92
RNA16K	16	0	0			-19507.37	23	39060.75	2231.58			-16956.12	26	33964.25	1393.19
RNA16B	15	0	0			-19544.50	22	39133.00	2303.83			-16966.04	25	33982.09	1411.03
