# Supplementary material for: An updated 18S rRNA phylogeny of tunicates based on mixture and secondary structure models
Source: BMC Evol Biol. 2009 Aug 5;9:187. doi: 10.1186/1471-2148-9-187 (PMC2739199; doi:10.1186/1471-2148-9-187)
Supplement: Additional file 2 — Species sampling, taxonomy and sequence accession numbers. The table indicate the taxonomy and the species sampling used in the present study with associated sequence Accession Numbers. The 30 new sequences obtained in this study are indicated with a star (*). [file 1471-2148-9-187-S2.pdf]

Taxonomy	Species	Accession no.
**Urochordata**		
Class Ascidiacea		
Order Aplousobranchia		
Family Clavelinidae		
	Clavelina meridionalis	FM244840*
	Pycnoclavella aff. *detorta*	FM244841*
Family Didemnidae		
	Cystodytes sp.	FM244842*
	Didemnum molle	AB211071.1
	Didemnum sp. *AB211072*	AB211072.1
	Didemnum sp. *AB211073*	AB211073.1
	Diplosoma mitsukurii	AB211096.1
	Diplosoma ooru	AB211097.1
	Diplosoma simile	AB211101.1
	Diplosoma simileguwa	AB211107.1
	Diplosoma sp. *AB211116*	AB211116.1
	Diplosoma sp. *AB211121*	AB211121.1
	Diplosoma virens	AB211109.1
	Leptoclinides madara	AB211070.1
	Lissoclinum badium	AB211078.1
	Lissoclinum bistratum	AB211079.1
	Lissoclinum patella	AB211085.1
	Lissoclinum punctatum	AB211086.1
	Lissoclinum sp.	AB211095.1
	Lissoclinum timorense	AB211092.1
	Trididemnum paracyclops	AB211074.1
Family Polycitoridae		
	Distaplia dubia	AB211068.1
	Eudistoma gilboviride	AB211069.1
Family Polyclinidae		
	Aplidium pliciferum	AB211067.1
Order Phlebobranchia		
Family Ascidiidae		
	Ascidia ahodori	AB104871
	Ascidia ceratodes	L12378
	Ascidiella sp.	FM244843*
	Phallusia fumigata	FM244844*
	Phallusia mammillata	AF236803
	Phallusia nigra	FM244845*
Family Cionidae		
	Ciona intestinalis	AB013017
	Ciona savignyi	AB191429
Family Corellidae		
	Chelyosoma siboja	AB104872
	Corella inflata	AY903930
	Corella eumyota	FM244846*
Family Perophoridae		
	Ecteinascidia herdmanni	FM244847*
	Ecteinascidia turbinata	FM244848*
	Perophora sagamiensis	AB104873
	Perophora viridis	FM244849*
Family Octacnemidae		
	Megalodicopia hians	AB075543
Order Stolidobranchia		
Family Molgulidae		
	Molgula arenata	AY903919
	Molgula bleizi	L12418
	Molgula citrina	L12420
	Molgula complanata	L12422
	Molgula manhattensis	L12426
	Molgula occidentalis	FM244850*
	Molgula occulta	L12430
	Molgula oculata	L12432
	Molgula pacifica	AY040738
	Molgula provisionalis	L12434
	Molgula pugetiensis	AY903920
	Molgula retortiformis	AY903921
Family Pyuridae		

	Boltenia villosa	AY903924
	Halocynthia igaboja	AY903925
	Halocynthia roretzi	AB013016
	Halocynthia spinosa	FM244851*
	Herdmania curvata	AF165827
	Herdmania momus	X53538
	Herdmania sp.	FM244852*
	Microcosmus polymorphus	FM244853*
	Microcosmus sabatieri	FM244854*
	Microcosmus squamiger	FM244855*
	Pyura dura	FM244856*
	Pyura gangelion	FM244857*
	Pyura haustor	AY903926
Family Styelidae		
	Botrylloides violaceus	AY903927
	Botryllus planus	DQ346653
	Botryllus schlosseri	AB211066
	*Botryllus schlosseri**	FM244858*
	Cnemidocarpa finmarkiensis	L12413
	Cnemidocarpa humilis	FM244859*
	Dendrodoa grossularia	L12416
	Metandrocarpa taylori	AY903922
	Pelonaia corrugata	L12440
	Polycarpa mytiligera	FM244860*
	Polycarpa papillata	DQ346654
	Polycarpa pomaria	L12441
	Styela gibbsii	AY903923
	Styela montereyensis	L12443
	Styela plicata	L12444
	Symplegma viride	DQ346655
Class Thaliacea		
<u>Order Doliolida</u>		
	Doliolum denticulata	FM244861*
	Doliolum nationalis	AB013013
<u>Order Pyrosomida</u>		
	Pyrosoma atlanticum	AB013011
	Pyrosoma godeauxi	FM244862*
	Pyrosomella verticillata	FM244863*
<u>Order Salpida</u>		
	Cyclosalpa quadriluminis	FM244864*
	Ihlea racovitzai	FM244865*
	Salpa cylindrica	FM244866*
	Salpa thompsoni	FM244867*
	Thalia democratica	D14366
Class Appendicularia		
Family Oikopleuridae		
	Megalocercus huxleyi	FM244868*
	Oikopleura dioica	AB013014
	Oikopleura labradoriensis	FM244869*
	Oikopleura sp.	D14360
**Outgroups**		
Echinodermata		
	Antedon serrata	D14357
	Asterias amurensis	D14358
	Strongylocentrotus purpuratus	L28055
Hemichordata		
	Balanoglossus carnosus	D14359
	Ptychodera flava	AF278681
	Saccoglossus pusillus	AF236800
Cephalochordata		
	Branchiostoma floridae	M97571
Vertebrata		
	Anolis carolinensis	AY859624
	Chrysemys sp.	AY859627
	Danio rerio	BX296557
	Gallus gallus	AF173612
	Homo sapiens	X03205
	Petromyzon marinus	M97575
	Raja schmidti	AF278682
	Xenopus laevis	X04025
