# Supplementary figures and images for: An updated 18S rRNA phylogeny of tunicates based on mixture and secondary structure models
Source: BMC Evol Biol. 2009 Aug 5;9:187. doi: 10.1186/1471-2148-9-187 (PMC2739199; doi:10.1186/1471-2148-9-187)

**A**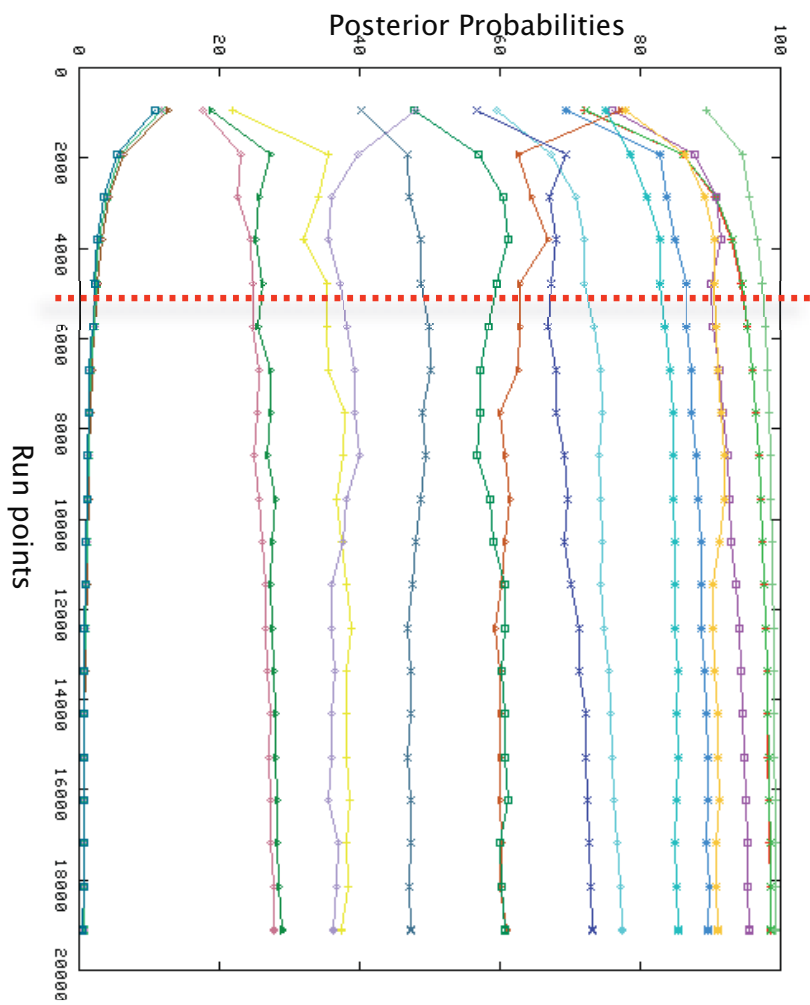**B**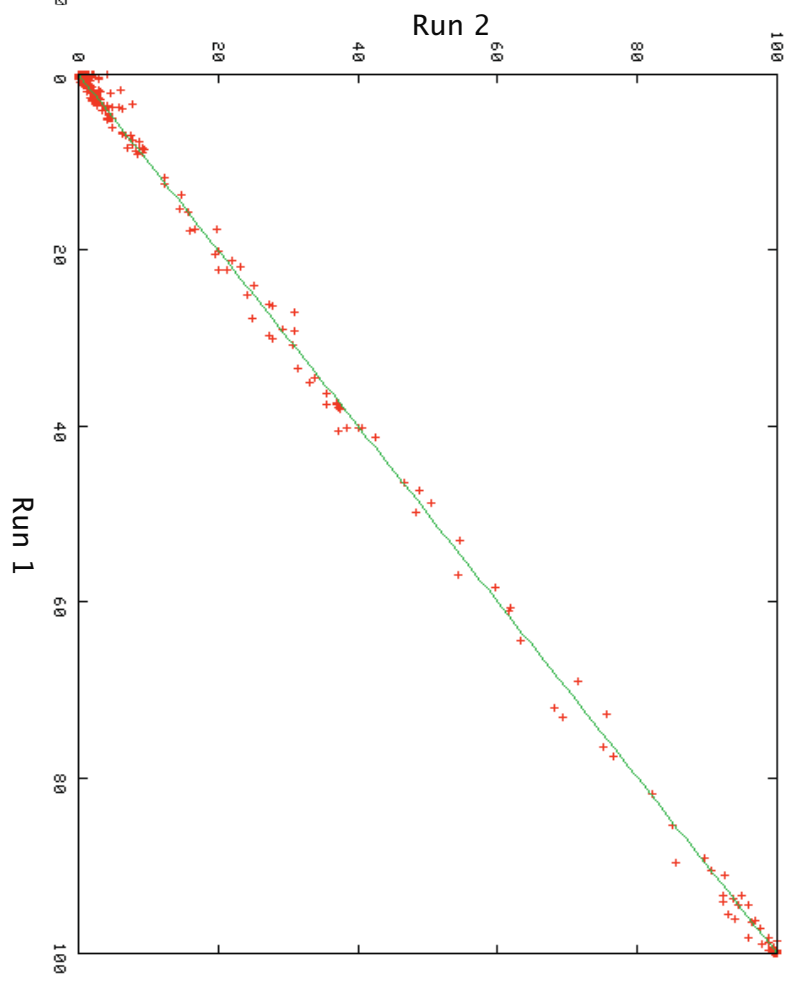

Supplement: Additional file 3 — Monitoring the convergence of MCMC in Bayesian analyses. The figure illustrates the post-analysis of chain convergence in Bayesian analyses under the CAT-GTR+Γ4 model for the 88-taxon dataset, using the AWTY system (Nylander et al. 2007). A. Cumulative plot of clade posterior probabilities of the 20 more variable splits over a run of 20,000 cycles (i.e. 1,500,000 MCMC generations) sampled at every cycle. The vertical red line indicates the determined burn-in value of 5,000. B. Comparisons of clade posterior probabilities between the two independent MCMC runs. [file 1471-2148-9-187-S3.pdf]
